# Prognostic value of gender and primary tumor location in metastatic colon cancer

**DOI:** 10.7150/jca.85748

**Published:** 2023-09-04

**Authors:** Antonino Grassadonia, Erminia Carletti, Antonella De Luca, Patrizia Vici, Francesca Sofia Di Lisa, Lorena Filomeno, Giuseppe Cicero, Laura De Lellis, Serena Veschi, Rosalba Florio, Davide Brocco, Pietro Di Marino, Saverio Alberti, Teresa Gamucci, Paola Borrelli, Alessandro Cama, Nicola Tinari

**Affiliations:** 1 Department of Innovative Technologies in Medicine and Dentistry, and Center for Advanced Studies and Technology (CAST), G. D'Annunzio University Chieti-Pescara, 66100 Chieti, Italy.; 2 Department of Medical, Oral and Biotechnological Sciences, and Center for Advanced Studies and Technology (CAST), G. D'Annunzio University Chieti-Pescara, 66100 Chieti, Italy.; 3 Unit of Phase IV Trials, IRCCS Regina Elena National Cancer Institute, 00144 Rome, Italy.; 4 Department of Surgical, Oncological and Oral Sciences, Section of Medical Oncology, University of Palermo, 90133 Palermo, Italy.; 5 Department of Pharmacy, G. D'Annunzio University, Chieti-Pescara, 66100 Chieti, Italy.; 6 Clinical Oncology, S.S. Annunziata Hospital, 66100 Chieti, Italy.; 7 Unit of Medical Genetics, Department of Biomedical Sciences-BIOMORF, University of Messina, 98125 Messina, Italy.; 8 Medical Oncology, Sandro Pertini Hospital, 00159 Rome, Italy.; 9 Laboratory of Biostatistics, Department of Medical, Oral and Biotechnological Sciences, G. D'Annunzio University Chieti-Pescara, 66100 Chieti, Italy.

**Keywords:** Metastatic colorectal cancer (mCRC), Gender, Tumor location, RAS status, Prognosis

## Abstract

Sex might influence prognosis in patients affected by colorectal cancer. We retrospectively studied a cohort of patients affected by metastatic colon cancer (mCC) stratified by sex and primary tumor location. RAS mutational status was also included in the analysis. Overall, 616 patients met the eligibility criteria, 261 women and 355 men. Neither gender, nor RAS mutational status influenced overall survival (OS) in the entire population. As expected, patients with right-sided colon cancer (RCC) had a significant shorter OS compared to those with left-sided colon cancer (LCC) (21.3 *vs* 33.1 months, p= 0.002). When the analysis was performed stratifying for gender, RCC retained worse prognosis among men (OS 20.5 *vs* 33.9 months, p= 0.008), but not among women (p= 0.132). Similarly, the presence of RAS mutations had no prognostic effect in women, but was significantly associate with shorter survival in men (OS 29.5 *vs* 33.7 months, p= 0.046). In addition, when comparing clinical outcome of women or men according to sidedness and RAS mutational status, RCC was associated with dismal prognosis only in men with RAS mutated tumor (OS 17.2 *vs* 32.3 months, p= 0.008). Our study highlights the importance of gender in the outcome of patients with mCC.

## Introduction

Colorectal cancer is the third cause of cancer-related death in the world, after lung and prostate cancer in men and after lung and breast cancer in women 1. In the US, the 5-year survival rate is 90% for people with localized stage, but falls to 14% for those with metastatic disease 2. Modern first line chemotherapeutic regimens, with the addition of monoclonal antibodies against EGFR (Cetuximab or Panitumumab) in RAS wild-type (wt) tumors or monoclonal antibodies against VEGF (Bevacizumab) in RAS mutated (mut) tumors have improved patient overall survival (OS) 3.

In recent years, epidemiologic studies have revealed that right-sided colon cancers (RCC), i.e. tumors occurring in the cecum, ascending colon or hepatic flexure, are characterized by poorer prognosis compared to left-sided colon cancers (LCC), i.e. tumors of the splenic flexure, descending, sigmoid and rectosigmoid colon 4-6. Studies on the molecular features of colorectal cancer in relation to primary tumor location have showed important differences: high microsatellite instability (MSI-H), BRAF mutations, and cytosine-guanosine (CpG) island methylation phenotype (CIMP) are frequently observed in RCC 7,8, while chromosomal instability and mutations in the TP53 and APC genes are more common in LCC 9. In addition, RCC presents a molecular pattern associated with intrinsic resistance to EGFR inhibition as compared with LCC 10-12, and a clear benefit from anti‐EGFR therapies has been reported only in patients with tumors originating in the left side of the colon 13,14. Consistently, in a previous retrospective study we have observed a more favorable outcome in patients with LCC, but not in those with RCC, treated with anti-EGFR agents compared to those who received Bevacizumab 15. On the contrary, Bevacizumab significantly improved survival in patients with RCC 16, indicating a predominant involvement of pro-angiogenetic factors in the tumors originating from the right side of the colon.

Along with sidedness, gender may influence colorectal cancer outcome. Compared to women, men seem to have a worse survival rate 17-19. Levels of circulating estrogens 20, oral contraceptives 21, hormonal replacement therapy 22, diet 23, physical activity 24, and microbiome diversity 25 have been proposed as factors responsible for reduced colorectal cancer incidence and death in women. Nevertheless, compared to men, women are more frequently diagnosed with the more aggressive right-sided proximal tumor 9. The reason for this difference is not known, but it is plausible the existence of a different biology of RCC in the two sexes. Thus, clinical outcome of women and men with RCC could be different compared with their peers with LCC.

The present study was carried out to investigate gender-associated survival differences in a cohort of patients affected by metastatic colon cancer (mCC) in relation to RAS mutational status and the anatomic location of the primary tumor.

## Patients and Methods

### Study design and data collection

Patients with newly diagnosed mCC consecutively referred to five Italian cancer centers between January 2010 and December 2020 for first-line therapy were included in this study. For each patient, gender, age, and baseline clinical-pathological features, including tumor histotype, tumor grade, site of metastasis, number of metastatic sites, primary tumor location, RAS mutational status, ECOG performance status, were collected. Information on previous adjuvant chemotherapy, surgery for primary tumor and/or metastasis, were recorded as well. Patient candidates for supportive care after the diagnosis of mCC were excluded from the study.

### Clinical Assessment

Overall survival (OS) data were analyzed in the entire population and after stratification of patients by gender, primary tumor location and the presence or absence of RAS mutations. OS was defined as the time from therapy initiation to death or last annotation on clinical records. The date of study cutoff was December 15, 2020.

### Statistical Analysis

Descriptive analysis was carried out using mean ± standard deviation or median and interquartile range (IQR) for the quantitative variables and percentages values for the qualitative ones.

Normality distribution was assessed by the Shapiro-Wilk test. Survival analysis was performed by applying the Kaplan-Meier estimator and Log-rank test for equality of survivor functions. The association with clinical features was analyzed with univariate Cox model of proportional hazards (Hazard Ratio - HR and 95% CI) for OS, and the applicability assumption was evaluated by the Schoenfeld test. A p value ≤ 0.05 was retained as the limit of statistical significance. The SPSS version 15.0 statistical software was used to perform all the analyses.

## Results

### Patients' characteristics

Overall, 616 patients were consecutively diagnosed with mCC and treated with first-line chemotherapy in the five participating Institutions, and were included in the survival analyses. Their clinical and pathological characteristics are summarized in Table 1.

The median age was 66 years (IQR 58-73) and the majority of patients were male (57.6%). Median age was similar in women and men, 64 years and 67 years, respectively. The primary tumor was left-sided in 403 (65.4%) patients, right-sided in the remaining 213 (34.6%). There was a slightly higher prevalence of RAS wt tumors (351 patients, 57%). Most patients, 418 (67.9%), did not received adjuvant chemotherapy after the first diagnosis of colon cancer, 510 (82.8%) had surgery of the primary tumor, and 155 (25.2%) had surgery of metastasis. Three hundred and twenty-three (52.4%) patients presented with multiple metastases. A good performance status (ECOG 0 or 1) was present in 93.4% of cases.

Importantly, all the reported characteristics, including location of primary tumor, were well balanced between men and women, with the exception of RAS mut tumors that were more frequent in women (48% *vs* 39%, p= 0.023). RAS mut tumors resulted also significantly higher in RCC compared to LCC, 51% and 39%, respectively (p= 0.005) (Table 2), but were equally distributed between the two sex in relation to the sidedness of primary tumor.

Even treatment approach was similar in the two sexes: independently from gender and tumor location, 70% of patients with RAS wt tumors received anti-EGFR agents (Cetuximab or Panitumumab) in association with the standard chemotherapy backbone, while most patients with RAS mut tumors (71%) received Bevacizumab. Table 3 summarizes the treatment choice in first and second line according to RAS mutational status in the entire cohort.

### Overall Survival

**Figure 1:**
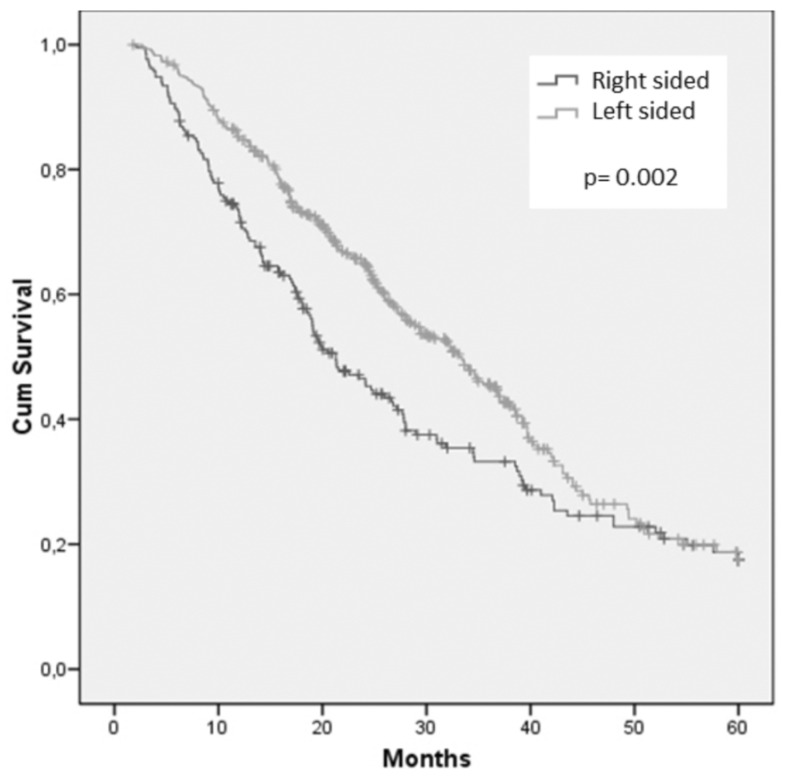
Cumulative overall survival of the whole population stratified by sidedness of primary tumor.

After a median follow-up of 21.4 months, 390 (63.3%) patients were dead. Overall survival was significantly affected by primary tumor location. Median OS was 21.3 months for patients with RCC and 33.1 months for those with LCC (Figure 1).

Neither gender nor RAS mutational status showed prognostic impact in the entire population. Median overall survival (OS) of patients stratified by gender and divided according to tumor sidedness and RAS mutational status are summarized in Table 4.

**Figure 2:**
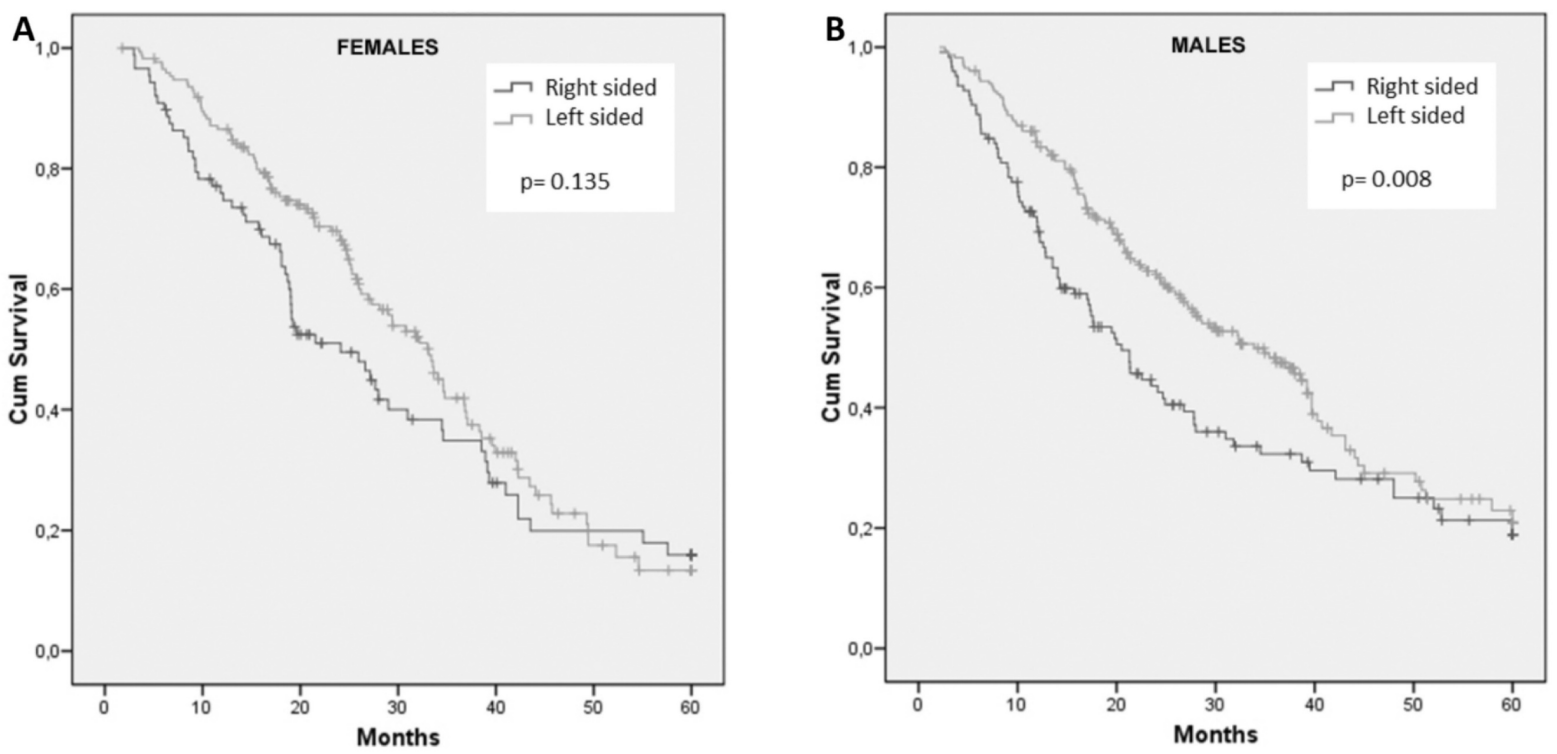
Cumulative overall survival of female (A) and male (B) patients stratified by sidedness of primary tumor.

Frequencies of right- and left-sided tumors were comparable between male and female patients. For female patients, survival did not differ with regard to primary tumor site (HR: 1.28; 95% CI, 0.93 to 1.76; p = 0.135) (Figure 2A). In contrast, tumor sidedness influenced survival among male patients. In fact, men with right-sided tumors showed a significantly inferior OS compared to those with left-sided tumor, 20.5 months and 33.9 months, respectively (HR: 1.45; 95% CI, 1.10 to 1.92; p= 0.008) (Figure 2B).

Similarly, mutation of RAS did not influence survival among women, while was significantly associated with worse prognosis in men (p= 0.046) (Table 4).

**Figure 3:**
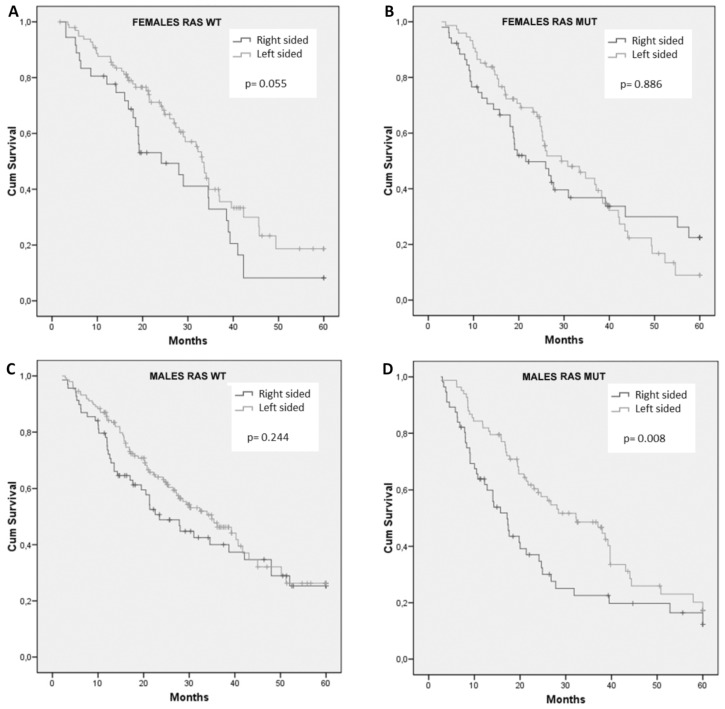
Cumulative overall survival of females with RAS wild-type (A) or RAS mutant (B) tumors and males with RAS wild-type (C) or RAS mutant (D) tumors stratified by sidedness of primary tumor

When considering survival according to sidedness separately in the group of patients with RAS wt or in those with RAS mut, again no difference was observed among women (Figure 3A and 3B), with a trend toward worse survival in RCC of the RAS wt group (HR: 1.58; 95% CI, 0.98 to 2.53; p = 0.055). Interestingly, men with RCC had a significant reduced survival only in RAS mut tumors (Figure 3D vs 3C). In particular, in male sex, patients with RAS mut tumors had a median OS of only 17.2 months in RCC compared to 32.2 months in LCC (HR: 1.73; 95% CI, 1.15 to 2.60; p = 0.008) (Figure 3D).

## Discussion

The results of this retrospective study are in line with the evidence that RCC, compared to LCC, is significantly associated with reduced survival, emphasizing the importance of primary tumor location in defining the prognosis of patients affected by mCC. However, our study revealed that RCC-associated poor outcome is observed in men rather than in women and that this sex difference is particularly evident in RAS mut tumors, indicating a negative impact of RAS mutations in men affected by tumor arising in the right side of the colon.

Despite a lower incidence of RCC 9, men with mCC have a general worse prognosis than women 17-19. In our cohort median OS was similar in the two sexes, in agreement with other studies that failed to demonstrate differences in survival between men and women 26,27. The lack of consensus has been attributed to a confounding hormonal effect that is not considered in the different studies, including menopausal status and hormonal replacement therapies 28.

RAS mutations have been reported in 40-50% of mCC and, in some studies, were more frequently observed in RCC than LCC 29 and more often in females than males 30. Consistently, in our cohort, the incidence of RAS mut tumors was 43% and was significantly higher among tumors of the right side of the colon and in females. RAS mutations have been showed to predict a dismal prognosis in patients with mCC 31,32, mainly in the presence of G12C or G12S variants 33, and particularly in patients treated with bevacizumab 34-36. In our study, RAS mut tumors displayed a worse prognosis, but only in men. The interpretation of this result will be discussed below, on the light of a possible increased effectiveness of bevacizumab in women with RCC.

Concerning sidedness, our findings emphasize the poor outcome of patients with RCC, compared to those with LCC, as reported in several clinical studies 4-6. The aggressive behavior of RCC has been attributed to a higher occurrence of BRAF mutations 37, PIK3CA mutations 38-40 or CpG island methylation phenotype (CIMP) 41-43, all factors associated with shorter survival 44-47. However, thus far, no study has focused on sex disparities relative to RCC.

In the present study we report a worse prognosis for men with RCC, when comparing survival separately in the two sexes according to tumor sidedness. This finding strongly suggests the existence of a different biology of RCC in men and women. In some way, adverse prognostic factors could negatively influence the course of cancer arising in the right side of colon in men, but not in woman. However, at the best of our knowledge, no such evidence has been reported yet.

Recently, a unique metabolic phenotype has been described in RCC of women, but not in LCC of women neither in colon cancer of men in both locations 48. In particular, it has been demonstrated that female RCC displays a high ATP-consuming metabolism supplied by oxidation of fatty acids rather than glycolysis. The increased ATP production seems to be directed to the synthesis of asparagine that is used by tumor cells as an amino acid exchange factor, leading to increased intracellular levels of threonine and serine. These two amino acids, in turn, determine mTOR activation that is eventually responsible for tumor growth and invasiveness 48. On the bases of this mechanism we would expect that the general poor prognosis of RCC would be even worse in women. Instead, we observed the opposite: compared to LCC, the survival in RCC was shorter only in men.

We could try to explain this apparent paradox by assuming a possible different activity of the anti-angiogenic agent bevacizumab in RCC in the two sexes. This hypothesis is based on the above-described mechanism responsible for mTOR activation which is exclusively expressed in RCC of women 48. In fact, an increased activity of mTOR is a well-known mechanism promoting angiogenesis 49, and the presence of tumor neo-angiogenesis is a well-established predictive factor of response to bevacizumab 50,51. In this scenario we could hypothesize that tumors arising in the right side of the colon in women express a pro-angiogenic phenotype and, for this reason, would be more sensitive to bevacizumab. On the contrary, tumors arising in the left side of the colon in woman, as well as those arising in either side of the colon in men, do not have this pro-angiogenic phenotype and, therefore, are less responsive to bevacizumab. Thus, given the intrinsic worse prognosis of RCC, the higher efficacy of bevacizumab in women would counterbalance the basic adverse prognostic factors acting in right-sided tumors. As a result, we did not find difference in survival between LCC and RCC in women. In men, on the contrary, the lack of such a benefit from bevacizumab in RCC resulted in a better outcome for LCC.

In addition, we showed that this gender disparity was more pronounced in patients with RAS mut tumors. In this subgroup, the negative prognostic impact of RAS mutations was clearly evident in males, in spite of a higher incidence of RAS mutations in women. Again, we could interpret this result assuming that women may receive a greater benefit from bevacizumab in RCC, as discussed above, and considering that bevacizumab has been prevalently administered in patients with RAS mut tumors compared to those with RAS wt, who preferentially received anti-EGFR agents. Thus, in a sample enriched with bevacizumab-treated patients, i.e. patients with RAS mut tumors, males showed a shorter survival compared to females and, as expected in this subgroup, male with RCC showed the worst survival curve when the analysis was performed to compare left versus right tumors.

Overall, the findings presented herein may coherently be interpreted by an increased anti-tumor activity of bevacizumab in women with RCC. Notably, data have been provided indicating that the addition of bevacizumab to chemotherapy determines a clinical benefit only in right-sided tumors 16 and that RCC, but not LCC, is endowed with a pro-angiogenic microenvironment characterized by increased expression of endothelial nitric oxide synthase (eNOS), COX2, and ephrin type‐B receptor 4 (EPHB4) 52. We suggest that these evidences would be influenced by the higher incidence of RCC in women who are the patients who may benefit the most from anti-angiogenic treatments due to the unique metabolic phenotype of cancer arising in this side of the colon.

With the limitations of a retrospective study, the possibility that co-morbidities would have influenced the clinical course of the disease, and the impossibility to evaluate hormonal interference due to lack of information, in our database, about personal medical history, menopausal status, history of hormone replacement therapy, and use of contraceptive agents, the findings we presented are enough robust given the large sample size and the homogenous therapeutic approach for all patients, thus far not influenced by gender nor by tumor sidedness.

Prospective analyses of patient's outcome in relation to gender, sidedness, and RAS mutational status, along with the determination of tumor genetic, epigenetic and metabolic alterations, could help in the near future to identify the different molecular portrait of RCC and LCC, opening to potential novel therapeutic agent to be specifically used for women or for men. We strongly recommend to take sex into account in future colorectal cancer research.

## Conclusions

Primary tumor location and gender are important prognostic/predictive factors to consider in studies on mCC. In the present study we found that men affected by tumors arising in the right side of the colon have a shorter survival compared to those with left-sided tumors, especially in the presence of RAS mut. No such difference was observed among women.

### Author Contributions

Conceptualization, A.G. and N.T.; methodology P.B.; formal analysis, P.B., S.V. and R.F.; resources, F.S.D.L. and L.F.; data curation, D.B., P.D.M. and A.D.L.; writing—original draft preparation, A.G. and E.C.; writing—review and editing, A.C., P.V., G.C. and N.T.; visualization, L.D.L. and D.B.; supervision, S.A., T.G. and N.T. All authors have read and agreed to the published version of the manuscript.

### Institutional Review Board Statement

This study was conducted according to the guidelines of the Declaration of Helsinki and approved by the Institutional Review Board of Chieti-Pescara on 04 Feb 2016 (IRB No. 01 2016-02-04).

### Informed Consent Statement

Patient consent was waived due to the retrospective nature of the study and the anonymous clinical data used in the analysis.

### Data Availability Statement

Data will be made available from the corresponding author on reasonable request.

## Figures and Tables

**Table 1:** Characteristics of the whole population.

Variable	N = 616	%
**Median age, ys (IQR)^1^**	66 (58-73)	
**Age (years)**		
≤ 65	301	48.9
> 65	315	51.1
**Gender**		
Female	261	42.4
Male	355	57.6
**Sidedness**		
Left	403	65.4
Right	213	34.6
**Grade**		
G1	5	0.8
G2	386	62.7
G3	140	22.7
Unknown	85	13.8
**ECOG PS**		
0	357	58.0
1	218	35.4
2	38	6.20
3	3	0.4
**Ras Staus**		
Wild-type	351	57.0
Mutated	265	43.0
**Previous adjuvant chemo**		
No	418	67.9
Yes with oxaliplaton	121	19.6
Yes w/out oxaliplatin	77	12.5
**Number of metastasis**		
1	293	47.6
>1	323	52.4
**Surgery of primary tumor**		
No	106	17.2
Yes	510	82.8
**Surgery of metastasis**		
No	461	74.8
Yes	155	25.2

^1^ IQR, Interquartile Range

**Table 2:** Distribution of RAS mutational status according to sidedness and sex.

Variable	N (%)	Sidedness					Sex				
		Left (%) (N= 403)	Right (%) (N= 213)	p-value			Male (N= 355)		Female (N= 261)		p-value
**RAS Status**				**0.005**						**0.023**	
Wild-type	351 (57)	246 (61)	105 (49)			216 (61)		135 (52)			
Mutated	265 (43)	157 (39)	108 (51)			139 (39)		126 (48)			
											

**Table 3:** Type of therapy administered in relation to RAS mutational status.

Variable	N (%)	RAS Status		
		Wild-type (%) (N= 351)	Mutated (%) (N= 265)	p-value
** I line chemotherapy**				**0.020**
+ Bevacizumab	251 (41)	62 (18)	189 (71)	
+ Anti-EGFR*	245 (40)	245 (70)	0 (0)	
Chemo alone	120 (19)	44 (12)	76 (29)	
** II line chemotherapy**				0.517
+ Anti-VEGF°	169 (27)	91 (26)	78 (29)	
+ Anti-EGFR*	72 (12)	72 (21)	0 (0)	
Chemo alone	239 (39)	116 (32)	123 (46)	
Supportive care	136 (22)	72 (21)	64 (25)	

*Not included in the statistical analysis. °Including Bevacizumab or Aflibercept

**Table 4:** Overall survival in female and male patients according to tumor sidedness and RAS status.

Variable	Male (N= 355)					Female (N= 261)			
	N (%)	Median (mo)	HR (95% CI)	p-value		N (%)	Median (mo)	HR (95% CI)	p-value
**Sidedness**									
Left	230 (65)	33.9	1.00			173 (66)	33.1	1.00	
Right	125 (35)	20.5	1.45 (1.10-1.92)	**0.008**		88 (34)	24.1	1.28 (0.93-1.76)	0.135
**RAS Status**									
Wild-type	216 (61)	33.7	1.00			135 (52)	31.6	1.00	
Mutated	139 (39)	29.5	1.30 (1.01-1.70)	**0.046**		126 (48)	31.2	1.05 (0.77-1.43)	0.760
Sidedness in RAS wild-type*									
Left	147 (68)	35.0	1.00			99 (73)	33.1	1.00	
Right	69 (32)	23.5	1.25 (0.86-1.84)	0.244		36 (27)	24.1	1.58 (0.98-2.53)	0.055
**Sidedness in RAS mutated°**									
Left	83 (60)	32.3	1.00			74 (59)	29.4	1.00	
Right	56 (40)	17.2	1.73 (1.15-2.60)	**0.008**		52 (41)	21.5	1.03 (0.66-1.61)	0.886

*Percentage (%) is referred to patients with RAS wild-type tumor °Percentage (%) is referred to patients with RAS mutated tumor
